# Antiproliferative Properties of Scandium Exopolysaccharide Complexes on Several Cancer Cell Lines

**DOI:** 10.3390/md19030174

**Published:** 2021-03-23

**Authors:** Javier Muñoz-Garcia, Mattia Mazza, Cyrille Alliot, Corinne Sinquin, Sylvia Colliec-Jouault, Dominique Heymann, Sandrine Huclier-Markai

**Affiliations:** 1 Institut de Cancérologie de l’Ouest, Université de Nantes, Blvd Jacques Monod, F-44805 Saint-Herblain, France; javier.munoz@ico.unicancer.fr (J.M.-G.); dominique.heymann@univ-nantes.fr (D.H.); 2 GIP ARRONAX, 1 rue Aronnax, CEDEX 3, F-44817 Nantes, France; mattia.mazza@subatech.in2p3.fr (M.M.); alliot@arronax-nantes.fr (C.A.); 3 Laboratoire SUBATECH, 4 rue Alfred Kastler, BP 20722, CEDEX 3, F-44307 Nantes, France; 4 Centre de Recherche en Cancérologie et Immunologie Nantes Angers, INSERM, U892, 8 quai Moncousu, CEDEX 1, F-44007 Nantes, France; 5 IFREMER, Institut Français de Recherche pour L’exploitation de la mer, rue de l’Ile d’Yeu, BP21105, CEDEX 3, F-44311 Nantes, France; corinne.sinquin@ifremer.fr (C.S.); Sylvia.Colliec.Jouault@ifremer.fr (S.C.-J.); 6 Department of Oncology and Metabolism, Medical School, University of Sheffield, Sheffield S10 2TN, UK

**Keywords:** exopolysaccharides, scandium, theranostic, cancer cell lines, proliferation

## Abstract

Antimetastatic properties on both murine and human osteosarcoma cell lines (POS-1 and KHOS) have been evidenced using exopolysaccharide (EPS) derivatives, produced by *Alteromonas infernus* bacterium. These derivatives had no significant effect on the cell cycle neither a pro-apoptotic effect on osteosarcoma cells. Based on this observation, these EPSs could be employed as new drug delivery systems for therapeutic uses. A theranostic approach, i.e., combination of a predictive biomarker with a therapeutic agent, has been developed notably by combining with true pair of theranostic radionuclides, such as scandium ^47^Sc/^44^Sc. However, it is crucial to ensure that, once complexation is done, the biological properties of the vector remain intact, allowing the molecular tropism of the ligand to recognize its molecular target. It is important to assess if the biological properties of EPS evidenced on osteosarcoma cell lines remain when scandium is complexed to the polymers and can be extended to other cancer cell types. Scandium-EPS complexes were thus tested in vitro on human cell lines: MNNG/HOS osteosarcoma, A375 melanoma, A549 lung adenocarcinoma, U251 glioma, MDA231 breast cancer, and Caco2 colon cancer cells. An xCELLigence Real Cell Time Analysis (RTCA) technology assay was used to monitor for 160 h, the proliferation kinetics of the different cell lines. The tested complexes exhibited an anti-proliferative effect, this effect was more effective compared to EPS alone. This increase of the antiproliferative properties was explained by a change in conformation of EPS complexes due to their polyelectrolyte nature that was induced by complexation. Alterations of both growth factor-receptor signaling, and transmembrane protein interactions could be the principal cause of the antiproliferative effect. These results are very promising and reveal that EPS can be coupled to scandium for improving its biological effects and also suggesting that no major structural modification occurs on the ligand.

## 1. Introduction

The key role of glycosaminoglycans (GAGs), natural polysaccharides found in mammalian tissue, has been recognized in the regulation of cell properties and functions. They are now considered as pharmacological targets to treat several diseases and notably metastatic cancers. GAGs play a central role in the organization of the extracellular matrix (ECM) through their binding to various molecules (ECM components, cell-surface receptors, growth factors and cytokines). Nowadays, several therapeutic strategies are developed with the aim of restoring cell function due to a degradation of endogenous GAGs either in protecting endogenous GAGs or in using exogenous GAGs or GAG-mimetics [1,2,3,4].

Unfractionated heparin and its low-molecular-weight (LMW) derivatives are widely used to prevent or treat venous thromboembolism, which is a common complication in cancer patients. Clinical trials have shown that cancer patients treated with heparins have improved, with a decrease in cancer progression and a longer survival time [5,6], which may be explained by heparin’s functional interference with critical biological steps of metastasis spread [7]. Despite their therapeutic potential, side-effects induced by their anticoagulant properties (e.g., hemorrhagic complications and heparin-induced thrombocythemia) may occur, limiting thus a long-term treatment. Heparin could present also a contamination with prion proteins or oversulfated chondroitin sulfate [8]. Heparin’s use in therapy is sometimes limited due to this hazard profile. As a result, the number of studies on heparin mimetic molecules has increased in recent years. Polysaccharides may be a new source of heparin-like molecules among the possible drug candidates. Traditional algal or modern bacterial polysaccharides may be used as a substitute for mammalian GAGs [9]. Furthermore, preliminary animal studies have shown that polysaccharides extracted from eukaryotes and prokaryotes living in the marine environment could reveal a better benefit/risk ratio [9]. Algal polysaccharides like fucoidans–especially low-molecular-weight fucoidan preparations–have previously been shown to have heparin-like properties with low hemorrhagic risks [10]. As soluble molecules or polymers, marine polysaccharides derived from bacteria have a lot of promise in cell therapy and tissue engineering. When compared to other polysaccharides from eukaryotes, they can be produced in bioreactors under completely regulated conditions [11].

Among these marine polysaccharides, an exopolysaccharide (EPS) of interest was isolated from the culture broth of a hydrothermal bacterium called *Alteromonas infernus*. A monosulfated nonasaccharide composed of six neutral hexoses and three hexuronic acids, including one galacturonic acid unit bearing one sulfate group, makes up the repeating unit of this EPS. It presents a high molecular weight (>10^6^ g mol^−1^) and a low sulfate content (<10%) [12,13,14]. When compared to heparin, this native EPS had a very poor anticoagulant effect. To improve its biological activities and provide a GAG mimetic compound, native EPS has been modified first by radical depolymerization, then by over-sulfation to create EPS derivatives [15,16]. In the following, EPS-DR denotes native EPS that has undergone radical depolymerization, whereas EPS-DRS denotes EPS that has been further over-sulfated. These two chemical modifications allow for a decrease in molecular weight while increasing sulfate content [17]. The anticoagulant efficacy of this LMW highly sulfated EPS derivative is increased over that of its native precursor, but it is still 10 and 5 times less potent than unfractionated and LMW heparin, respectively. Following the oversulfation stage, an oversulfated EPS structure (EPS-DRS) has been suggested, with three additional sulfate groups on both glucose and galactose constitutive of the repeating unit (unpublished data). It is made up of three hexose units that have been oversulfated and have been reported elsewhere [15,16,17].

In vitro studies on bone remodeling have displayed different levels of bone resorption regulation by EPS-DRS, most of them leading to pro-resorptive effects [15]. These results have brought to consider those compounds to be tested on osteosarcoma, characterized by high potency to induce lung metastasis [18]. EPS-DRS of different sizes have been tested against two osteosarcoma cell lines (mouse POS-1 and human HOS) revealing that an EPS derivative of 15 kDa could effectively inhibit both migration and invasiveness of osteosarcoma cells in vitro, while it could be very efficient at inhibiting the establishment of lung metastases in vivo [19].

Since their natural tropism in targeting malignant tumor and their metastasis, EPS have been recently considered as vector to be combined with theranostic radionuclide pairs. Through the possibility of doing therapy and diagnostic at the same time, theranostic compounds represent new innovative clinical tools to be employed in a perspective of “personalized/precision” medicine [20]. More precisely, a theranostic pair ^44^Sc/^47^Sc could be both employed for Positron Emission Tomography (PET) imaging to detect primary and secondary tumors, as well as for Targeted RadioTherapy in order to bombard tumor tissues with gamma radiations [21]. Complexation between EPS and scandium has already been assessed and quantified in a previous work [22]. The EPS derivatives selected for this study are a highly sulfated one, called DRS (M_w_ = 27 kDa) and a lower-sulfated one, called DR (with M_w_ = 16 kDa). The complexation properties of these two EPS with scandium were higher than the ones on glucuronic and galacturonic acids, even though Sc-EPS complexes appear to be less stable (*K*_ScEPS-DR_ = 9.14; *K*_ScEPS-DRS_ = 7.69) than scandium complexes with 1,4,7,10-Tetraazacyclododecane-1,4,7,10-tetraacetic acid (DOTA) and diethylenetriaminepentaacetic acid (DTPA), classically used in radiopharmaceutical drugs.

For further use as a therapeutic vector, it is important to ensure that EPS may keep the ability of targeting cancer cells even when complexed with scandium. For this reason, in vitro studies concerning both cell proliferation and cell viability of various tumor cell lines were realized here to evaluate if there is a synergetic effect of Sc-EPS complexes on different cancer cell lines, compared to the EPS alone and scandium alone. To this aim, the cell index of human osteosarcoma (MNNG/HOS), human melanoma (A375), human lung cancer (A549), human glioblastoma (U251), and human breast cancer (MDA231) were monitored over a week using XCELLigence^®^ technology. XCELLigence measures the electric impedance of cell assessed. This impedance is directly related to the cell adhesion process and, consequently, to cell proliferation and/or cell death. Moreover, the effect of the metal-to-ligand ration of the EPS-Sc complexes was assessed and, a heparin was used as a reference system for these EPS.

## 2. Results

### 2.1. Human MNNG/HOS Osteosarcoma Cell Line

#### 2.1.1. Effect of Scandium Alone on Cell Proliferation and Viability

**Figure 1:**
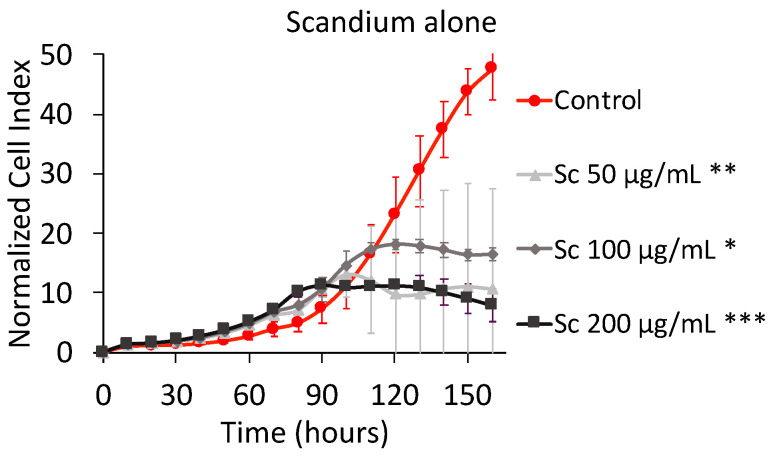
Normalized cell index plot of MNNG/HOS cells after 160 h of contact with Sc^3+^ at 50 µg mL^−1^ (**), 100 µg mL^−1^ (*), and 200 µg mL^−1^ (***) * = *p* value ≤ 0.0001, ** = *p* value ≤ 0.005, *** = *p* value ≤ 0.05.

Figure 1 shows the effect of scandium alone on MNNG/HOS osteosarcoma cell proliferation as well as cell viability. Concerning the cell index (CI) measured by xCELLigence RTCA, the software employed transformed the CI of all wells at all time points to the normalized cell index (NCI), which was considered the CI value of individual wells at the same time point (base-time). This transformation made the NCI more comparable between the wells [23].

The NCI plot shown in Figure 1 depicts two different kinds of curves. The ascending bi-exponential curve of the control is due to the fact that, in its well, the space to grow up was not saturated yet. The saturation was reflected by a plateau. Normally, two scenarios can occur once the plateau is set: (1) the plateau continues until starting a definitive descending evolution characteristic of damaged cells that are not attached to the surface of the well; (2) the plateau continues until starting a momentary decrease followed by a brief rise, that is characteristic of the replacement of damaged cells by new healthy cells. The curves for scandium concentrations at 50 and 100 µg mL^-1^ showed a plateau that reflected no more MNNG/HOS cells proliferation but the detachment from the well was not observed yet. For the scandium concentration at 200 µg mL^-1^, the detachment of cells slightly began at 140 h. Unfortunately, the time frame of 160 h was not sufficient to see if the decrease was definitive or kept oscillating. The MTT assay confirmed that the concentration of 200 µg mL^−1^ displayed a reduction of cell viability of around 25% after 160 h. For lower concentrations (i.e., 50 µg mL^−1^ and 100 µg mL^−1^), an increase of cell viability was noticed.

#### 2.1.2. Effect of EPS and Heparin Alone

**Figure 2:**
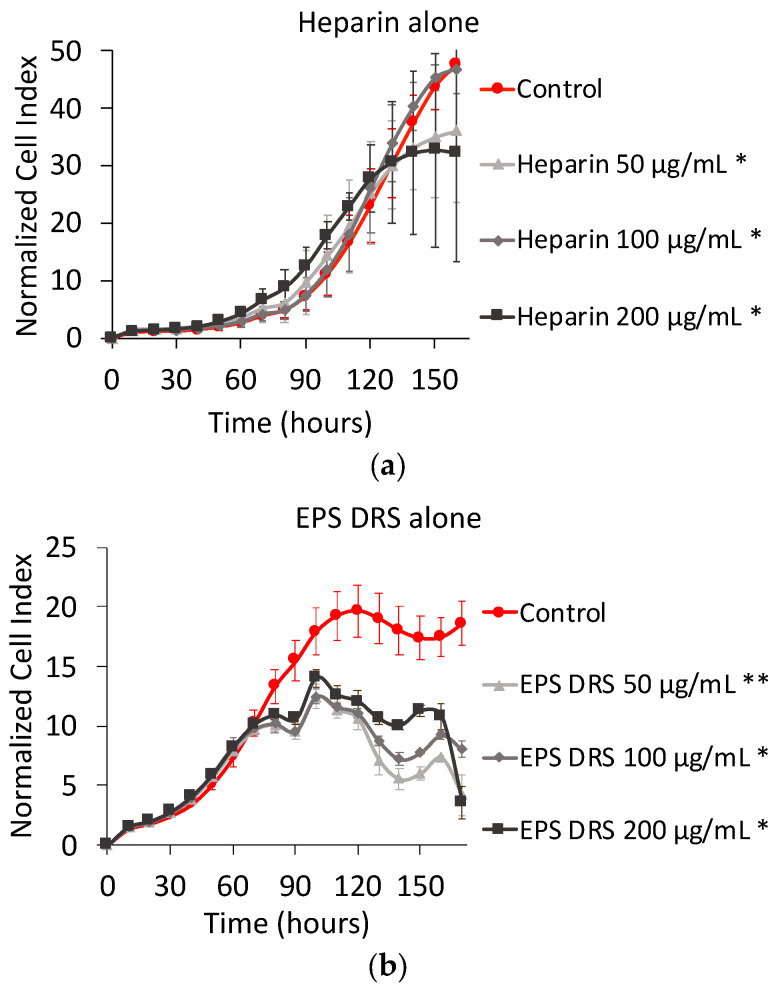

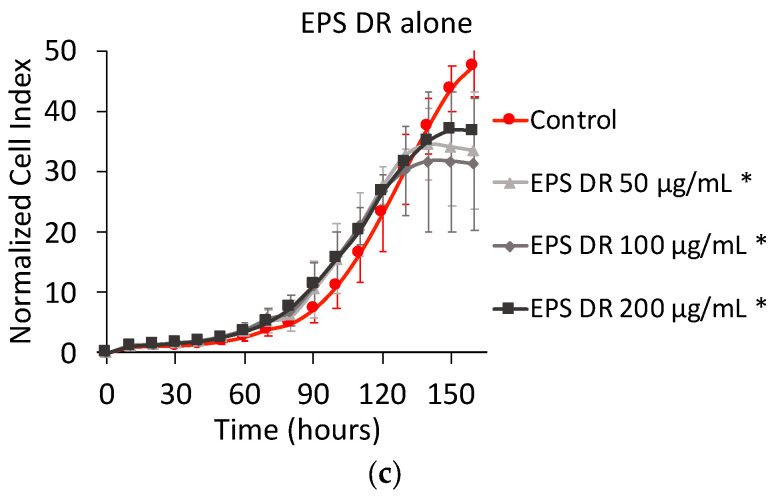
To the left, normalized cell index plot of MNNG/HOS cells after 160 of contact with (**a**) heparin alone at 50 µg mL^−1^ (*), 100 µg mL^−1^ (*), and 200 µg mL^−1^ (*); (**b**) EPS DRS alone at 50 µg mL^−1^ (**), 100 µg mL^−1^ (*), and 200 µg mL^−1^ (*); (**c**) EPS-DR alone at 50 µg mL^−1^ (*), 100 µg mL^−1^ (*), and 200 µg mL^−1^ (*).

The effect of both EPS-DR and -DRS and heparin on cell proliferation and cell viability is showed in Figure 2. It is noticed that NCI plot of EPS-DRS is very different from the ones of heparin and EPS-DR. For EPS-DRS, a second round of xCELLigence experiment was performed, leading to a higher number of MNNG/HOS cells (i.e., 5000 cells) compared to the first one (i.e., 3000 cells). This is why the control of EPS-DRS reached the peak of proliferation sooner than the two other ones, while for heparin and EPS-DR cells were still proliferating. This allowed evidencing the effect of the not modulated by the addition of heparin whatever the concentration considered. It was noticed that for 50 µg mL^−1^ of EPS-DR, the lowest sulfated EPS of this study, 100% viability was reached compared to the control.

#### 2.1.3. Effect of Polysaccharide:Scandium Complexes

##### Heparin:Scandium (Hep:Sc) Complex

**Figure 3:**
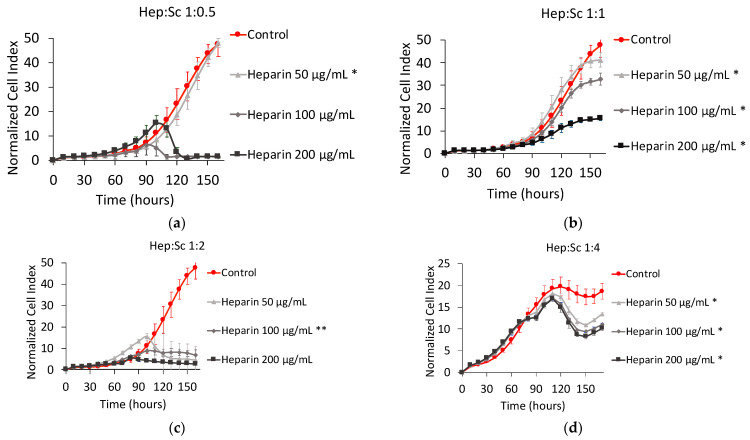
Normalized cell index Plot of MNNG/HOS cells after 160 h of contact with different stoichiometric ratios L:M: (**a**) heparin:scandium (Hep:Sc) 1:0.5 at 50 µg mL^−1^ (*), 100 µg mL^−1^, and 200 µg mL^−1^; (**b**) Hep:Sc 1:1 at 50 µg mL^−1^ (*), 100 µg mL^−1^ (*), and 200 µg mL^−1^ (*); (**c**) Hep:Sc 1:2 at 50 µg mL^−1^, 100 µg mL^−1^ (**), and 200 µg mL^−1^; (**d**) Hep:Sc 1:4 at 50 µg mL^−1^ (*), 100 µg mL^−1^ (*), and 200 µg mL^−1^ (*).* = *p* value ≤ 0.0001, ** = *p* value ≤ 0.005.

Figure 3 shows the NCI plot from xCELLigence RTCA of MNNG/HOS cells after 160 h of contact. The Hep:Sc complexes were prepared for four different metal-to-ligand ratios. For a metal-to-ligand ratio of 1:0.5 (Figure 3a), with a total concentration of heparin of 50 µg mL^−1^, there was no effect on cell proliferation. After 100 h of contact, for the same metal-to-ligand ratio but with higher concentrations of heparin (i.e., 100 and 200 µg mL^−1^ respectively), there was a significant decrease of the cell proliferation rates.

With a metal-to-ligand ratio Hep:Sc of 1:1 (Figure 3b), the decrease in cell proliferation was well displayed and dose-dependent. For metal-to-ligand ratio of Hep:Sc of 1:2 (Figure 3c), there was no further exponential growth after 90 h of contact for all the concentrations of heparin considered. Finally, for a metal-to-ligand ratio of Hep:Sc of 1:4 (Figure 3d), there were the same proliferation rates between the control and treated cells until 100 h of incubation followed by a temporary decrease of cell proliferation.

If those results are compared to the ones obtained on cell viability, the metal-to-ligand ratio ratios of 1:0.5 and 1:4 exhibited higher percentages of cell viability. In contrast, for a constant metal-to-ligand ratio of 1:1, a significant decrease of almost 40% is observed when increasing the total concentration of heparin. The only drop on cell viability is observed for a metal-to-ligand ratio of 1:2 and for a concentration of heparin of 200 µg mL^−1^; at these conditions, there is no more viable cells present in the well after 160 of contact.

##### EPS-DRS:Scandium (EPS-DRS:Sc) Complex

**Figure 4:**
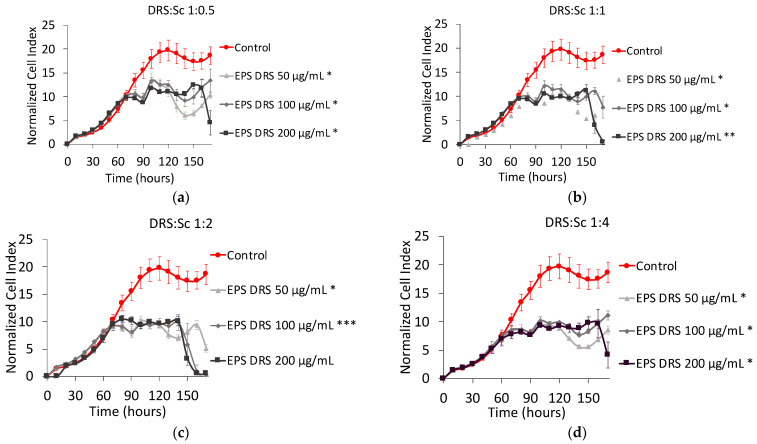
Normalized cell index plot of MNNG/HOS cells after 160 h of contact with different stoichiometric ratios L:M: (**a**) EPS-DRS:Sc 1:0.5 at 50 µg mL^−1^ (*), 100 µg mL^−^1 (*), and 200 µg mL^−1^ (*); (**b**) EPS-DRS:Sc 1:1 at 50 µg mL^−1^ (*), 100 µg mL^−1^ (*), and 200 µg mL^−1^ (**); (**c**) EPS-DRS:Sc 1:2 at 50 µg mL^−1^ (*), 100 µg mL^−1^ (***), and 200 µg mL^−1^; (**d**) EPS-DRS:Sc 1:4 at 50 µg mL^−1^ (*), 100 µg mL^−1^ (*), and 200 µg mL^−1^ (*).* = *p* value ≤ 0.0001, ** = *p* value ≤ 0.005, *** = *p* value ≤ 0.05.

Figure 4 shows the NCI plot of MNNG/HOS cells after 160 h of contact with four metal-to-ligand ratio of EPS-DRS:Sc complex. Regarding the four metal-to-ligand ratio of the EPS-DRS:Sc complexes, the shape are very close to each other, meaning that neither the metal to ligand ratio, nor the total concentration of EPS-DRS play a key role in the cell proliferation. Only one difference could be noticed, a slight reduction of cell proliferation, in the range 10–15 to 5–10 of NCI. For an EPS-DRS concentration of 200 µg mL^−1^, the cell proliferation decreases suddenly after 150 h, after than a plateau has been reached. This has been noticed for the four metal-to-ligand ratios considered. This seems stopping the oscillating kinetics to very low and stable NCI values, comparable to those of heparin complexes (see Figure 3). No significant changes in cell viability for the four metal-to-ligand ratios considered, was observed, even at for the concentration of 200 µg mL^−1^ in EPS-DRS.

##### EPS-DR:Scandium (EPS-DR:Sc) Complex

**Figure 5:**
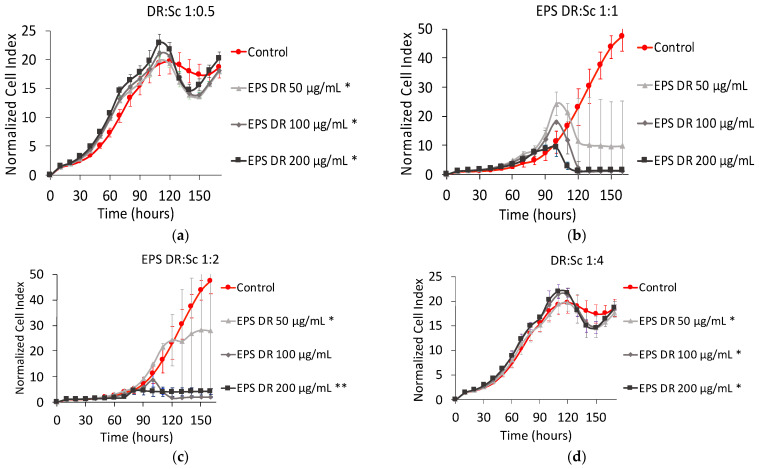
Normalized cell index plot of MNNG/HOS cells after 160 h of contact with different stoichiometric ratios L:M: (**a**) EPS-DR:Sc 1:0.5 at 50 µg mL^−1^ (*), 100 µg mL^−1^ (*), and 200 µg mL^−1^ (*); (**b**) EPS-DR:Sc 1:1 at 50 µg mL^−1^, 100 µg mL^−1^, and 200 µg mL^−1^; (**c**) EPS-DR:Sc 1:2 at 50 µg mL^−1^ (*), 100 µg mL^−1^, and 200 µg mL^−1^ (**); (**d**) EPS-DR:Sc 1:4 at 50 µg mL^−1^ (*), 100 µg mL^−1^ (*), and 200 µg mL^−1^ (*). * = *p* value ≤ 0.0001, ** = *p* value ≤ 0.005.

Figure 5 shows the NCI plot on MNNG/HOS cells after 160 h of contact with four metal-to-ligand ratios of EPS-DR:Sc complex. For the ratios EPS-DR:Sc 1:1 and 1:2, the cell proliferation profiles are quite similar. For the metal-to-ligand ratio of 1:1, after 100 h of contact, a peak of proliferation was reached at very low NCI for all the EPS-DR concentrations considered. Then, there was a plateau that tended to zero NCI for the highest concentration of EPS-DR. For the ratios EPS-DR:Sc 1:1 and 1:2, the standard deviation of all the curves appeared very high. Likewise, even for metal-to-ligand ratio of 1:0.5 and 1:4, similar curves were obtained, that could be quite well superimposed with the control. For these two metal-to-ligand ratios, the cell proliferation started oscillating after 120 h.

The cell proliferation profiles were compared to the cell viability results. For metal-to-ligand ratio of 1:0.5 and 1:4, there was a high percentage of cell viability after 160 h of contact whereas for metal-to-ligand ratio of 1:1, there was a significant decrease when increasing the concentration of EPS-DR. This behavior was very similar to the one observed on Hep:Sc complexes.

### 2.2. Human A375 Melanoma Cells

#### Effect of Scandium and Polysaccharides Alone

**Figure 6:**
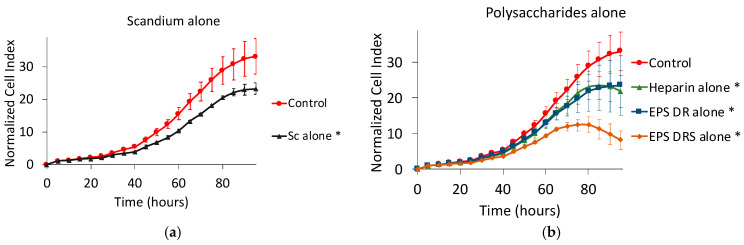
Normalized cell index plot of human A375 melanoma cells after 95 h of contact with (**a**) scandium at concentration of 100 µg mL^−1^ (*) and (**b**) polysaccharides alone at concentration of 100 µg mL^−1^: heparin (*), EPS-DRS (*), EPS-DR (*). * = *p* value ≤ 0.0001.

Figure 6 shows the effect on cell proliferation of scandium, as well as the three polysaccharides alone. All the compounds reduced the cell proliferation after 95 h of contact. The effect of scandium alone was comparable to that of heparin or EPS-DR alone. Nevertheless, the EPS-DRS alone, that had the higher sulfate content, displayed a more traditional profile of detaching cells.

### 2.3. Effect of Polysaccharide:Scandium Complexes

**Figure 7:**
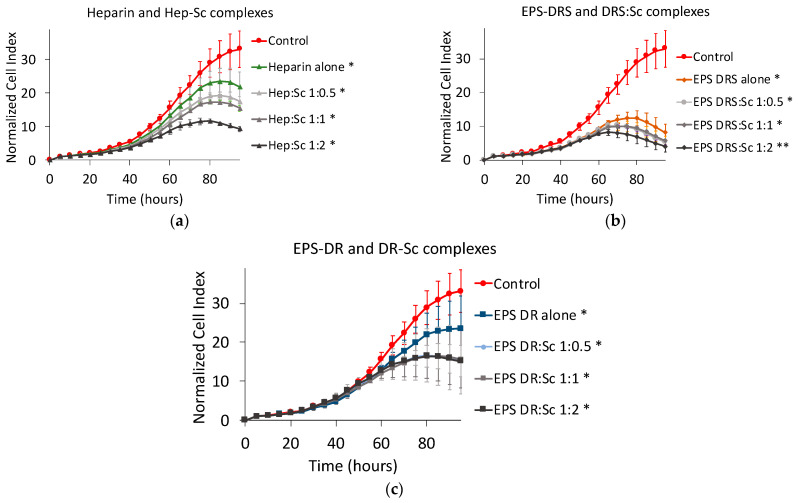
Normalized cell index plot of human A375 melanoma cells after 95 h of contact with different scandium-polysaccharide complexes at concentration of 100 µg mL^−1^ and various stoichiometric ratios: (**a**) Hep:Sc 1:0.5 (*), 1:1 (*), 1:2 (*), heparin alone (*); (**b**) EPS-DRS:Sc 1:0.5 (*), 1:1 (*), 1:2 (**), EPS-DRS alone (*); (**c**) EPS-DR:Sc 1:0.5 (*), 1:1 (*), 1:2 (*), EPS-DR alone (*). * = *p* value ≤ 0.0001, ** = *p* value ≤ 0.005.

For both EPS considered in this work, i.e., EPS-DR and -DRS, scandium-polysaccharide complexes were more efficient in reducing the cell proliferation than the EPS themselves as shown in Figure 7. For Hep:Sc (Figure 7a) and EPS-DRS:Sc (Figure 7b) complexes, at increasing metal-to-ligand ratio, a more important decrease in cell proliferation was observed. The same trend could be observed for EPS-DR:Sc complexes (Figure 7c). The best conditions for reducing the cell proliferation were reached for a metal-to-ratio of 1:2, leading to a cell proliferation reduction of approximately 55–88%.

### 2.4. Human A549 Lung Cancer Cells

#### 2.4.1. Effect of Scandium and Polysaccharides Alone

**Figure 8:**
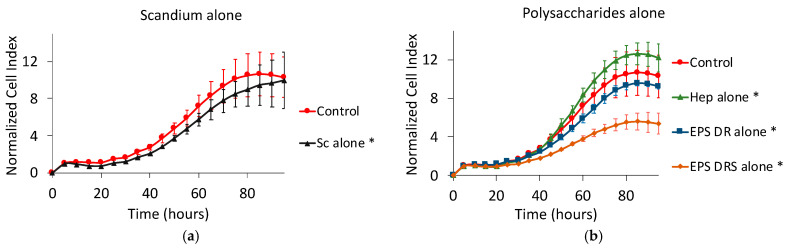
Normalized cell index plot of human A549 lung cancer cells after 95 h of contact with (**a**) scandium at concentration of 100 µg mL^−1^ (*) and (**b**) polysaccharides alone at concentration of 100 µg mL^−1^: heparin (*), EPS-DRS (*), EPS-DR (*). * = *p* value ≤ 0.0001.

From Figure 8a, scandium seemed having no impact on the proliferation of lung cancer cells after 95 h of contact. By contrast, among the polysaccharides considered in this work, only EPS-DRS seemed to slow down the kinetic of the cell proliferation. By comparison to EPS-DR, containing lower sulfate content, it could be assumed so far that the sulfate content of the macromolecule played a role on the cell proliferation, at least for the cell line A549.

#### 2.4.2. Effect of Polysaccharide:Scandium Complexes

**Figure 9:**
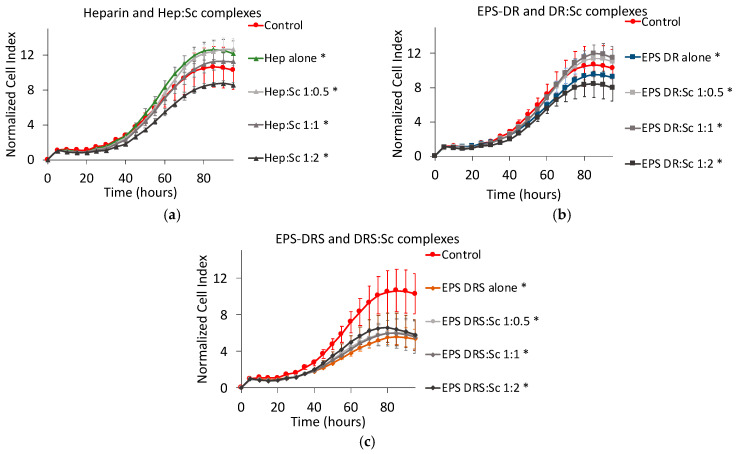
Normalized cell index plot of human A549 lung cancer cells after 95 h of contact with different scandium-polysaccharide complexes at concentration of 100 µg mL^−1^ and various stoichiometric ratios: (**a**) Hep:Sc 1:0.5 (*), 1:1 (*), 1:2 (*), heparin alone(*); (**b**) EPS-DR:Sc 1:0.5 (*), 1:1 (*), 1:2 (*), EPS-DRS alone (*); (**c**) EPS-DRS:Sc 1:0.5 (*), 1:1 (*), 1:2 (*), EPS-DR alone (*). * = *p* value ≤ 0.0001.

As shown in Figure 9a–c, the cell proliferation curves obtained when the cells contacted EPS-DR:Sc and Hep:Sc complexes, exhibited quite similar kinetic behaviors.

Only the metal-to-ligand ratio of 1:2 had an impact on the cell proliferation. This effect was more pronounced with EPS-DRS:Sc (Figure 9b) but for this one, there was no influence of the metal-to-ligand ratio.

### 2.5. Human U251 Glioblastoma Cells

**Figure 10:**
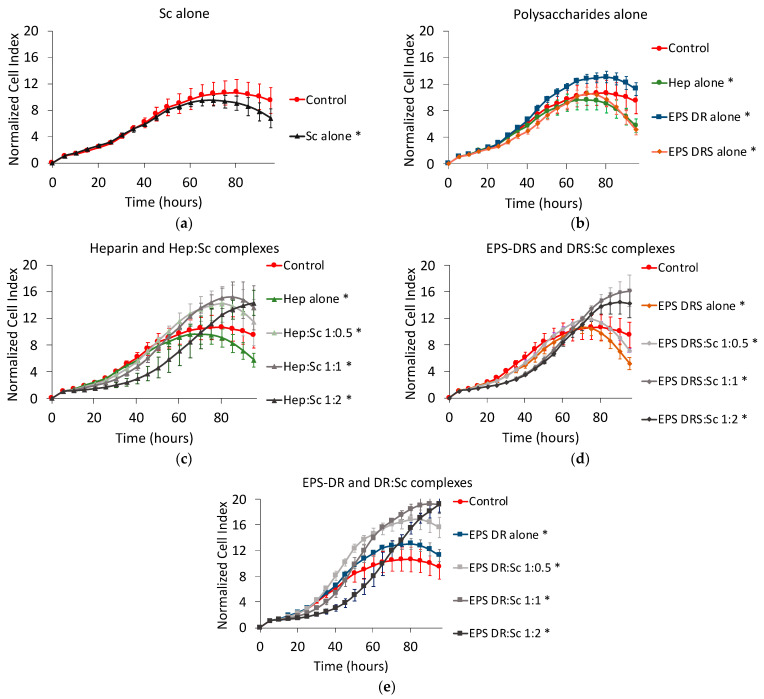
Normalized cell index plot of human U251 glioblastoma cells after 95 h of contact with (**a**) scandium at concentration of 100 µg mL^−1^ (*) and (**b**) polysaccharides alone at concentration of 100 µg mL^−1^: heparin (*), EPS-DRS (*), EPS-DR (*). Normalized cell index plot of U251 cells after 95 h of contact with different scandium-polysaccharide complexes at concentration of 100 µg mL^−1^ and various stoichiometric ratios: (**c**) Hep:Sc 1:0.5 (*), 1:1 (*), 1:2 (*), heparin alone(*); (**d**) EPS-DRS:Sc 1:0.5 (*), 1:1 (*), 1:2 (*), EPS-DRS alone (*); (**e**) EPS-DR:Sc 1:0.5 (*), 1:1 (*), 1:2 (*), EPS-DR alone (*). * = *p* value ≤ 0.0001.

Scandium alone induced a slight decrease of U251 cell proliferation (Figure 10a). Among the three polysaccharides, only heparin and EPS-DRS (Figure 10b), the most sulfated polysaccharides, slowed down the proliferation of approximately 45% compared to the untreated control group.

When considering the scandium complexes (Figure 10c–e), in particular, Hep:Sc and EPS-DR:Sc complexes showed a less efficient reduction in the proliferation of glioblastoma cells compared to the polysaccharide themselves. As observed for the other cell lines tested, there was an exception for the metal-to-ligand ratio 1:2 that influenced the kinetics. Additionally, EPS-DRS:Sc complexes were rather more efficient than DRS alone but this activity was not correlated to the metal-to-ligand ratio as has been evidenced by the other cell lines tested above.

### 2.6. Human MDA231 Breast Cancer Cells

**Figure 11:**
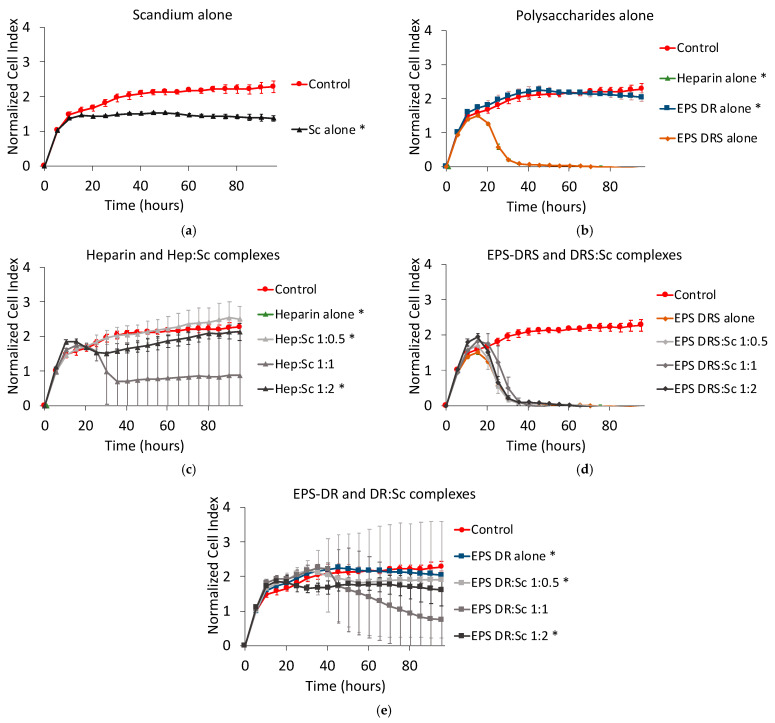
Normalized cell index plot of human MDA231 breast cancer cells after 95 h of contact with (**a**) scandium at concentration of 100 µg mL^−1^ (*) and (**b**) polysaccharides alone at concentration of 100 µg mL^−1^: heparin (*), EPS-DRS, EPS-DR (*). Normalized cell index plot of MDA231 cells after 95 h of contact with different scandium-polysaccharide complexes at concentration of 100 µg mL^−1^ and various stoichiometric ratios: (**c**) Hep:Sc 1:0.5 (*), 1:1, 1:2 (*), heparin alone (*); (**d**) EPS-DRS:Sc 1:0.5, 1:1, 1:2, EPS-DRS alone; (**e**) EPS-DR:Sc 1:0.5 (*), 1:1, 1:2 (*), EPS-DR alone (*). * = *p* value ≤ 0.0001.

Scandium alone slightly decreased the cell proliferation of breast cancer cell, as shown in Figure 11a. Among the polysaccharides alone, only EPS-DRS demonstrated highly reducing the kinetics of proliferation (Figure 11b).

Hep:Sc and EPS-DR:Sc complexes were more efficient than the respective polysaccharides alone only at the stoichiometric ratio L:M 1:1 (Figure 11c–e), but standard deviations were very high for all the curves. EPS-DRS:Sc complexes exhibited the same effect of the polysaccharide alone (Figure 11d).

### 2.7. Human Caco2 Colon Cancer Cells

**Figure 12:**
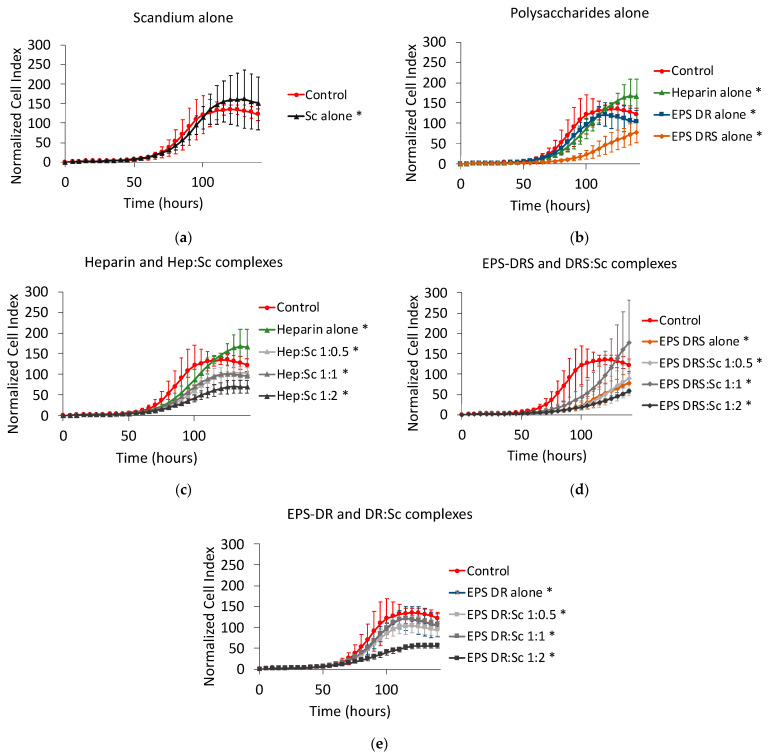
Normalized cell index plot of human Caco2 colon cancer cells after 140 h of contact with (**a**) scandium at concentration of 100 µg mL^−1^ (*) and (**b**) polysaccharides alone at concentration of 100 µg mL^−1^: heparin (*), EPS-DRS (*), EPS-DR (*). Normalized cell index plot of Caco2 colon cancer cells after 140 h of contact with different scandium-polysaccharide complexes at concentration of 100 µg mL^−1^ and various stoichiometric ratios: (**c**) Hep:Sc 1:0.5 (*), 1:1 (*), 1:2 (*), heparin alone (*); (**d**) EPS-DRS:Sc 1:0.5 (*), 1:1 (*), 1:2 (*), EPS-DRS alone (*); (**e**) EPS-DR:Sc 1:0.5 (*), 1:1 (*), 1:2 (*), EPS-DR alone (*). * = *p* value ≤ 0.0001.

For this cell line, the contact time was prolonged to 140 h to display significant effects of compounds. Scandium alone did not have any effect on cell proliferation of colon cancer cells, as shown in Figure 12a. Once again, only EPS-DRS demonstrated significantly reducing the kinetics of proliferation (Figure 12b).

Hep:Sc and EPS-DR:Sc complexes were more efficient than the respective polysaccharides alone only at the stoichiometric ratio of 1:2 (Figure 12c,e). EPS-DRS:Sc complexes exhibited the same effect of the polysaccharide alone (Figure 12d), except at the ratio 1:1.

Table 1 summarizes the effect of Hep:Sc and EPS:Sc complexes for different metal-to-ligand ratio and different concentrations on human osteosarcoma, melanoma, lung cancer, glioblastoma and breast cancer cell lines.

## 3. Discussion

### 3.1. Effect of Scandium Alone

Published data are quite scarce on scandium, which seems not having a major biological role. The food chain contains trace amounts of this metal, so the average daily intake per person is less than 0.1 µg [24]. To the best of our knowledge, the toxicity of scandium has not been reported. The major risk of scandium exposure could be aerosols and gasses that can be inhaled within a working environment (for instance rare-earth mining plants). A long-term exposure by inhalations may cause lung embolism. Kawai et al. [24] have reported that low concentrations of scandium can determine an overproduction of antibiotic when added to certain *Streptomyces* cultures.

In our study, a biological effect has been highlighted on the different cancer cell lines (osteosarcoma, lung, glioblastoma, melanoma, breast). The literature found on the evaluation of scandium has described an absorption and prolonged retention of scandium in some tissues [25], especially with accumulation in the liver, spleen and bone [26] or in the bone [27]. A complete biodistribution with ^46^Sc^3+^ [28] has been performed. From the best of our knowledge, there is no reported data on in vitro cytotoxicity studies of scandium with osteosarcoma cells. Only one study from Herath and Evans [29] described the effect of scandium oxide (Sc_2_O_3_), that is a solid compound, on a commercial human osteoblast-like cell line (TE85 HOS). Scandium oxide showed a lower cell proliferation after 3 weeks of contact compared to the control. TE85 HOS represent the parental cells from which the MNNG/HOS cells were derivated after chemical transformation with 0.01 µg mL^−1^ MNNG (a carcinogenic nitrosamine). TE85 HOS parental cell line is not tumorigenic in mouse in contrast to MNNG/HOS. Herath and Evans [29] also demonstrated by cell viability studies, that Sc_2_O_3_ did not affect cell viability.

### 3.2. Effect of Heparin and EPS Alone

It is already known that GAG participate in the regulation of several cellular events, such as cell adhesion, proliferation and migration [4]. Actually, GAGs are heterogeneous macromolecules characterized by a high diversity of disaccharide units that forms the primary structure. Major differences include types of uronic acid and hexosamine, number and position of *O*-sulfated and *N*-sulfated groups, resulting in GAGs with different chemical and biological properties [30]. Among GAGs, heparin has already showed a significant antiproliferative effect on various cell types [31,32,33]. Studies have demonstrated that high contents in heparin structure of iduronic acid (IdoA) and N-sulfated glucosamine (GLcN) are fundamental to inhibit cell proliferation [31]. The degree of sulfation has also revealed to be a critical factor in the inhibition of cell proliferation [31]. Nikitovic et al. [34] have proved a strong inhibition of cell proliferation of both normal and transformed osteoblasts by heparin already at low concentrations. In our study, heparin reduced, the cell proliferation in a dose-dependent manner after 160 h, as shown in Figure 2a. This time, however, is not enough to observe the drop of NCI like in EPS-DRS. The higher degree of sulfation could be the cause of such drop. These results agree with the previous in vitro studies relating the effect of these EPS on cell proliferation of MNNG/HOS cells [19] and osteoblastic cells [15].

It has been shown that high concentrations of GAGs interrupted growth factor-receptor signaling in osteoblasts-like Saos-2 cells by sequestering growth factors (e.g., FGF2) and preventing their interaction with cell-surface receptors [35]. This biological effect could be realized also with EPS, explaining the slowdown in cell proliferation kinetics.

Cell viability after 160 h of contact with the polysaccharides alone has been also assessed using MTT assays (results are not showed in this paper), and it resulted high for the polysaccharide concentrations considered. Considering the fact that the cell viability measured through the MTT assays, reflects mitochondrial metabolism as biochemical marker of cell viability, it could be reasonable to think that the slowdown or drop of cell proliferation is not imputable to the blockade of this biochemical process. EPS, as GAG mimetics, interact with cell transmembrane proteins such as selectins and integrins, responsible of cell adhesion and cell-cell interactions [36]. Since the decrease in proliferation kinetics reflects the detaching of cells from the gold biosensors in the well, it could be possible that the loss of adhesion could be derivate from the lack of normal functioning of these cell transmembrane proteins and not from an internal cell damage like the stop of metabolism.

In the literature there have been evidences on the role of heparin in lung cancer [37,38]. Low molecular weight heparins (LMWH) were found to have positive effects in decreasing the proliferation of metastasis through its anticoagulant and non-anticoagulant properties (inhibition of P- and L-selectins). The effects on cell proliferation on primary tumors are more contested since heparin and other GAGs do not seem to effectively reduce it [3,39]. By contrast, in the present study, an increase of proliferation of human lung cancer cells was clearly evidenced with heparin at the concentration of 100 µg mL^−1^ in Figure 11b, whereas EPS-DR and -DRS displayed a more moderate slowdown of the kinetics. It was evidenced also that the sulfation degree played a role on this proliferation kinetics. This increase of anti-proliferative effect with an increasing the degree of sulfation is in agreement with the results of Wright et al. [31].

LMWH have also been tested in the past on human melanoma cells displaying valid anti-proliferative effects [40]. Additional experiments on melanoma models have demonstrated a clear antimetastatic effect, attributable to the non-anticoagulant properties of heparin. The antimetastatic mechanism of that seems to reside in the inhibition of P- and L-selectins with consequent lack of cell-cell interactions [40]. In the present case, the different polysaccharides considered alone exhibited an anti-proliferative effect on the cell growth kinetics of human melanoma cells, revealing also that the degree of sulfation of these polysaccharides play a positive modulation on the proliferation by reducing it. These results agree with those showing another GAG [41], from animal environment, showing also a significant decrease in the proliferation of melanoma cells.

By contrast, the role of certain GAGs, such as chondroitin sulfate, is controversial in the pathogenesis of glioblastoma and breast cancer, since they seem enhance the tumor invasion [42,43]. The same stimulating effect on cell proliferation has been shown by heparin on colon cancer [44]. From the results presented in Figure 10b and Figure 12b, heparin and both EPS-DR and -DRS presented a slight effect in reducing the proliferation of glioblastoma and colon cancer cells. Schnoor et al. [45] have also demonstrated in vitro that heparin can cause a decrease of glioblastoma cell growth. Concerning breast cancer cell, no significant effect on the decrease of proliferation is showed, except for EPS-DRS. Comparing the effect of these three polysaccharides of interest, EPS DRS revealed to be the most efficient in reducing the proliferation kinetics of all the four cell lines tested, especially on MNNG/HOS cells where the effect is dose dependent.

### 3.3. Synergic Effect of Complexes

Polysaccharide-scandium complexes (EPS-DR:Sc, EPS-DRS:Sc and Hep:Sc) are more efficient than their respective polysaccharides alone in reducing the cell proliferation. That was the case for all the cell lines tested, thus revealing that the complexation with scandium does not prevent the biological activity of these polysaccharides. There is a synergetic effect by combining these polysaccharides with scandium, since the anti-proliferative effect has risen.

#### 3.3.1. Heparin:Sc (Hep:Sc)

On MNNG/HOS cell line, as shown in Figure 3, the complex Hep:Sc with a 1:0.5 metal-to-ligand ratio appears the most efficient complex in reducing the NCI to almost zero, followed by the metal-to-ligand ratio 1:2 of Hep:Sc. Both complexes exhibit a dose–response effect; in particular for the complex Hep:Sc with a 1:1 metal-to-ligand ratio. From a clinical point of view, the change in concentration of this complex would allow controlling its therapeutic window, revealing a good candidate for the elaboration of a new therapeutic strategy. The effect of the other complex seems to appear, instead, as an on-off effect, more difficult to control.

For the highest concentration for all the complexes tested on MNNG/HOS cells, the profile of their proliferation kinetics was quite different. The complexation with scandium and in particular, the different ratios seem to induce different effects. This could reside in a different polymer conformation assumed by heparin at different ratios of metal. From our previous work, the effect of added counterions on polysaccharide conformation has been assessed revealing how hydrodynamic volume and gyration radius of this polyelectrolyte is influenced by the ionic strength, so by the opposite charges provided by the presence of metal ions. If the conformation of the polysaccharide changes, the way it interacts with its macromolecular targets would change as well, resulting in a different modulation of the biological effect [45].

Considering a fixed concentration of Hep:Sc complexes of 100 µg mL^−1^, the increase of the metal to ligand ratio had an important effect also on human melanoma and colon cancer cell line. After 95 h of contact for melanoma cells (140 h for colon cancer cells), the ratio 1:2 seems to exhibit the most pronounced anti-proliferative effect, whereas the decrease in cell proliferation is clearly dependent on the L:M ratio. The change in conformation mediated by the complexation would positively modulate the biological effect. By contrast, the proliferation kinetic on human lung cancer, glioblastoma and breast cancer cells did not seem to be impacted by the complex. This behavior indicates that heparin is not efficient as anti-proliferative agent on primary tumors generated from these three human cancer cell lines.

#### 3.3.2. EPS-DRS:Sc and EPS-DR:Sc

Considering the effect of EPS-DR:Sc complexes on human osteosarcoma, melanoma cells, and colon cancer cells, the metal-to-ligand ratio of 1:1 and 1:2 revealed to have a significant impact on cell proliferation. One explanation of these two different kinetics may be related to the fact that complexation with scandium at these ratio lead into a change of conformation, this may inhibit cell proliferation for contact time superior to 90 h and at high concentrations of EPS. For metal-to-ligand ratio of 1:0.5 and 1:4, that are comparable, they show a very similar effect compared to EPS DR alone.

The same EPS-DR:Sc complexes did not display the same effect on human lung cancer, glioblastoma and breast cancer cell lines meaning that the slowdown in cell proliferation is not related to this specific kind of tumor. This seems in agreement with the fact that the biological effect normally influences only the formation of metastasis.

Glioblastoma appears to be not much sensitive to these GAG mimetics since even EPS-DRS:Sc did not show significant results in decreasing the proliferation kinetics. By the contrary, EPS-DRS:Sc revealed a great slowdown in the cell proliferation of human lung cancer, breast cancer, melanoma and colon cancer cell lines that was ratio dependent. The higher sulfate content of DRS may enhance the anticoagulant properties, resulting in a much stronger interaction with P- and L-selectins due the possibility to allow more electrostatic interactions with those proteins. Moreover, the possible change in conformation would act in a synergic way allowing a positive modulation of the anti-proliferative effect.

From cell assays, EPS-DRS:Sc with a 1:2 metal-to-ligand ratio, came out to be the best polysaccharide-scandium complex in reducing the cell proliferation of human osteosarcoma, human melanoma, human lung cancer, human breast cancer and colon cancer cells. These results confirm the importance of the degree of sulfation which positively enhances the cytostatic effect of these exopolysaccharides alone or complexed.

## 4. Materials and Methods

### 4.1. Molecules Assessed

Heparin sodium salt (*M*_w_ = 18,320 Da, S% = 10%) from porcine intestinal mucosa H4784 and scandium chloride hexahydrate were purchased from Sigma-Aldrich (Saint Quentin Fallavier, France). EPS derivatives DR (*M*_w_ = 16,440 Da, S% = 3%) and DRS (*M*_w_ = 27,360 Da, S% = 15%) were kindly provided by LEMMMB laboratory of IFREMER (Nantes). Polysaccharide solutions (heparin, EPS-DR and EPS-DRS alone) were prepared at high concentration stock solutions (2 × 10^−4^ mol L^−1^) in Milli-Q water; scandium alone solution was prepared at high concentration stock solution (10^−1^ mol L^−1^) in Milli-Q water. For each of the three polysaccharides, a set of complexes with four stoichiometric metal-to-ligand ratio (1:0.5, 1:1, 1:2, 1:4) has been prepared by mixing different aliquots of the previous prepared stock solutions. Higher stoichiometric ratios have been excluded to avoid cytotoxic effects related to high ionic strengths.

Complexation between scandium and polysaccharides has been previously checked and displayed using Free-Ion Selective Radiotracer Extraction (FISRE) as described somewhere else [45]. FISRE was used to determine the stability constants of a complex L:M, at tracer level, using a cationic exchange resin Chelex 100, poured into the solutions. These solutions contained fixed 10^−4^ mol L^−1^ Sc^3+^ and varying ligand concentration from 10^−9^ to 10^−3^ mol L^−1^. The resulting suspensions were daily monitored and adjusted to pH = 6. The separation of the solid and liquid phases was done by sedimentation. Aliquots of the supernatant from 0.4 to 1 mL were taken for ICP-AES analysis (ICP spectometer iCAP 6000 of Thermo Scientific, Illkirch, France).

M:L ratio has been established by preparing solutions where the concentration in mol/L of the metal was, from 0.5 to 4 times the one of ligand. After preparation, all 16 compounds (Sc alone, heparin alone and the four complexes Hep:Sc, EPS-DRS alone and the four complexes EPS-DRS:Sc, DR alone and the four complexes DR:Sc) were filtered with a 13 μm syringe filter before being frozen to prevent bacteria contamination. Fresh dilutions at 200, 100 and 50 μg/mL of each compound were obtained right before the experiments adding cell culture medium to aliquots from stock solutions.

### 4.2. Proliferation Assay

Human MNNG/HOS osteosarcoma cell line, A375 melanoma, A549 lung cancer, U251 glioblastoma, MDA231 breast cancer and Caco2 colon cancer cell lines were purchased from the American Tissue Cell Collection (ATTC, Molsheim, France). Human MNNG/HOS osteosarcoma cell line (ATCC) was cultured with DMEM high glucose, pyruvate, non-glutamine from Gibco (Thermo-Fisher), supplemented with glutamine (Thermo-Fisher) and 10% of fetal bovine serum (FBS, Thermo-Fisher). Human A375 melanoma cell line (ATCC) was cultured with DMEM 4.5 g/L high glucose, pyruvate, non-glutamine from Gibco (Thermo-Fisher), supplemented with glutamine (Thermo-Fisher) and 5% of fetal bovine serum (FBS, Thermo-Fisher). Human A549 lung cancer cell line (ATCC) was cultured with DEMEM/F12 (Sigma Aldrich), supplemented with glutamine (Thermo-Fisher) and 5% of fetal bovine serum (FBS, Thermo-Fisher). Human U251 glioblastoma cell line (ATCC) was cultured with DMEM 4.5 high glucose, pyruvate, non-glutamine from Gibco (Thermo-Fisher), supplemented with glutamine (Thermo-Fisher) and 5% of fetal bovine serum (FBS, Thermo-Fisher). Human MDA231 breast cancer cell line (ATCC) was cultured with L-15 media from Gibco (Thermo-Fisher), supplemented with glutamine (Thermo-Fisher) and 5% of fetal bovine serum (FBS, Thermo-Fisher). Human Caco2 colon cancer cell line (ATCC) was cultured with DMEM 4.5 g/L high glucose, pyruvate, non-glutamine from Gibco (Thermo-Fisher), supplemented with glutamine (Thermo-Fisher) and 5% of fetal bovine serum (FBS, Thermo-Fisher).

Cells were incubated at 37 °C with humidity saturated controlled atmosphere and 5% CO_2_. At confluence, cells are detached using Trypsin (Thermo-Fisher) and washed to neutralize the enzyme. MNNG/HOS cells were seeded in triplicate at 5000 cells per well (50 µL) with 50 µL of culture medium (to measure the background) for 4 h, the time necessary for cell seeding, in an E-Plate view 96 (Chem Agilent, Santa Clara, CA, USA) before adding 100 µL of compound at the three concentrations (50, 100 and 200 µg mL^−1^ for MNNG/HOS cells, 100 µg mL^−1^ for A375, A549, U251 cells). The choice of these concentrations provided a wide range to study a possible dose dependent response. Proliferation curves are normalized respect the time point of drug incorporation. The plate was monitored for 160 h (MNNG/HOS cells), 140 h (Caco2 cells) and 95 h (A375, A549, U251, MDA231 cells) using a RTCA instruments (Agilent and ACEA, Santa Clara, CA, USA). Experiments have been done in triplicates and repeated twice. Statistical tests have been conducted using Regression Data Analysis Tool in Excel^®^.

### 4.3. Viability Assay

MNNG/HOS cells were seeded in triplicate at 3000 cells par well (50 µL) with 50 µL of culture medium for 4 h in an E-Plate view 96 (Chem Agilent) before adding 100 µL of compound at the three concentrations (50, 100 and 200 µg mL^−1^). The choice of these concentrations provided a wide range to study a possible dose dependent response. The plate was left 160 h: the volume of each well was reduced to 100 µL before adding 10 µL of 5 mg/mL MTT (Sigma-Aldrich) and incubating for at least 3 h at 37 °C and 5% CO_2_. After this time, the liquid was removed and 200 µL of DMSO was added to each well to dissolve the formed formazan crystals before proceeding to the colorimetric quantification with a multi-well spectrophotometer (Victor 3x from PerkinElmer, Villebon-sur-Yvette, France) at the wavelength of 500–600 nm.

## 5. Conclusions

This in vitro study has demonstrated the biological effects of these unconventional exopolysaccharides. EPS derivatives (-DR or -DRS). EPS derivatives can slowdown cell proliferation kinetic, confirming their anti-proliferative effect on human osteosarcoma cells without significantly influencing the cell viability. Close results have been found also on human melanoma cells, while human glioblastoma cells have not been affected by those EPS derivatives. The anti-proliferative effect on other cell lines (i.e., human osteosarcoma, melanoma, breast cancer and lung cancer) is effective with polysaccharides but even more enhanced when EPS derivatives, and heparin, are complexed to scandium, in which the impact on the NCI is dose–response. The degree of complexation is represented by the different metal-to-ligand ratio. Different behaviors in the decrease of the proliferation kinetic have been evidenced depending on the metal-to-ligand ratio, that could be related to a conformation change of the polysaccharide when complexed with scandium. EPS-DRS:Sc with a 1:2 metal-to-ligand ratio has been shown to be the most effective compound in displaying a cytostatic effect. Our group has evidenced in a previous work [45] that the rheological behavior of EPS derivatives and heparin, notably the conformation, is influenced by the ionic strength of solvent, as well as at different concentrations of scandium. Thus, it is assumed that the change in conformation could modulate positively or negatively the antiproliferative effect. Concerning the cause of the antiproliferative or cytostatic effect, a damage in the cellular metabolism does not seem to be implicated since the cells remain viable, at least in the time frame of our study. The most probable explanation would be that an inhibition of growth factor-receptor signaling, as well as an interference of cell-cell adhesion mediated by specific transmembrane proteins.

These results are very encouraging in a theranostic perspective because of the intrinsic tropism of these EPS derivatives for cancer cells is maintained, even after the complexation. Additionally, the anti-proliferative effect on certain cell lines could be modulated by the degree of complexation. In the near future, the influence of the degree of complexation will be performed to investigate which are the best metal-to-ligand ratio in terms of efficacy. Besides, xCELLigence assay with EPS derivatives could be extended to other cell lines known to be sensible to GAG properties, while testing new assays to target other cellular mechanisms involved in cellular toxicity. Moreover, the biological effects of these EPS will be tested on normal cells as well in order to guarantee no cytostatic effect on healthy cells. The efficacy of these compounds will be evaluated with the PET tracer of ^44m/44^Sc.

## Figures and Tables

**Table 1:** Summary of the effect of Hep:Sc and EPS:Sc complexes for different metal-to-ligand ratio and different concentrations on MNNG/HOS, A375, A549, U251, MDA231 and Caco3. + = low anti-proliferative effect, ++ = medium anti-proliferative effect, +++ = high anti-proliferative effect, − = low proliferative effect, − − = medium proliferative effect, − − − = high proliferative effect, NT = not tested.

Complex	Ratio	Total Conc. of GAGs	Total Conc. of Sc	Effect on Cell Proliferation					
				MNNG/HOS	A375	A549	U251	MDA231	Caco2
	M:L	µg mL^−1^	µg mL^−1^	Osteosarcoma	Melanoma	Lung	Glioblastoma	Breast Cancer	Colon Cancer
Hep:Sc	1:0.5	50	0.36	No effect	NT				
		100	0.71	+ + +	+ +	−	−	No effect	+
		200	1.42	+ + +	NT				
	1:1	50	0.71	+					
		100	1.42	+ +	+ +	No effect	− −	+ +	+
		200	2.83	+ + +	NT				
	1:2	50	1.42	+ + +					
		100	2.83	+ + +	+ + +	+	− −	No effect	+ +
		200	5.66	+ + +	NT				
	1:4	50	2.83	+ +					
		100	5.66	+ +					
		200	11.32	+ +					
EPS-DRS:Sc	1:0.5	50	0.40	+ +					
		100	0.79	+	+ + +	+ +	+	+ + +	+
		200	1.58	+	NT				
	1:1	50	0.79	+ +					
		100	1.58	+ +	+ + +	+ +	− −	+ + +	−
		200	3.16	+ + +	NT				
	1:2	50	1.58	+ + +					
		100	3.16	+ + +	+++	+ +	−	+ + +	+ +
		200	6.31	+ + +	NT				
	1:4	50	3.16	+ + +					
		100	6.31	+ +					
		200	12.62	+ + +					
EPS-DR:Sc	1:0.5	50	0.24	No effect					
		100	0.48	No effect	+ +	−	− −	No effect	+
		200	0.95	No effect	NT				
	1:1	50	0.48	+ + +					
		100	0.95	+ + +	+ +	−	− − −	+ +	+
		200	1.90	+ + +	NT				
	1:2	50	0.95	+ +					
		100	1.90	+ + +	+ +	+	− − −	+	+ +
		200	3.79	+ + +	NT				
	1:4	50	1.90	No effect					
		100	3.79	No effect					
		200	7.58	No effect					

## Data Availability

Data available in a publicly accessible repository. The data presented in this study are openly available at [doi].

## Glossary

CI	cell index
EPS	exopolysaccharides
GAG	glycosaminoglycanes
LMW	low molecular weight
LMWH	Low molecular weight heparins
NCI	normalized cell index
RTCA	Real Cell Time Analysis
